# The Use of Biatain Ag in Hard-to-Heal Venous Leg Ulcers: Meta-Analysis of Randomised Controlled Trials

**DOI:** 10.1371/journal.pone.0067083

**Published:** 2013-07-02

**Authors:** David Leaper, Christian Münter, Sylvie Meaume, Alessandro Scalise, Nacho Blanes Mompó, Birte Petersen Jakobsen, Finn Gottrup

**Affiliations:** 1 Wound Healing Research Unit, Cardiff University, Cardiff, United Kingdom; 2 General Practitioner, Hamburg, Germany; 3 Hôpital Rothschild, Paris, France; 4 Ancona Politechnical University, Faculty of Medicine, Ancona, Italy; 5 Hospital de Manises, Valencia, Spain; 6 Coloplast A/S, Humlebaek, Denmark; 7 Copenhagen Wound Healing Center, Bispebjerg University Hospital, Copenhagen, Denmark; University Hospital Hamburg-Eppendorf, Germany

## Abstract

**Background:**

Venous leg ulcers are common, troublesome, and their failure to heal is often related to a heavy bio-burden. Ionized silver has both anti-inflammatory and antimicrobial properties. The ulcer healing properties of the silver releasing foam dressing Biatain Ag has been examined in 4 randomized controlled trials (RCTs).

**Aim:**

To evaluate ulcer healing through a meta-analytic approach after treatment with either Biatain Ag or a non-active dressing.

**Patients and Methods:**

685 subjects with pure or mixed hard-to-heal venous leg ulcers were included in the meta-analysis.

**Results:**

Biatain Ag showed a significant treatment effect (p<0.0001), responder rate (p<0.001), and healing rate (p = 0.002).

**Conclusion:**

The meta-analysis of the 4 RCTs provided statistical significant evidence to support the use of Biatain Ag dressing in treatment of hard-to-heal venous leg ulcers.

## Introduction

Chronic venous leg ulcers affect 1–3% of the adult population and account for the majority of lower extremity ulceration [1] and several studies have shown that more than 50% of leg ulcers have not healed after a year [2]–[4]. These ulcers may be delayed in having early appropriate treatment and may become recalcitrant to healing; they cause pain and suffering to patients, which impacts on their quality of life; and represent a significant financial burden on health systems.

Non-healing ulcers may express an inappropriate and excessive inflammatory phase of healing, usually related to a heavy or increasing bioburden of colonizing micro-organisms which stalls the healing process [5]; [6]. Antiseptics have been used in wound management in several forms for millennia. This has included the use of silver and more recently iodine, in its various forms, chlorhexidine and polyhexamethylene biguanide (PHMB) [7]–[12]. Antiseptic, including topical antimicrobials, can be toxic to healing tissue which has been shown in extensive experimental studies [13]; [14]. However the mode of action of antiseptics is different to that of antibiotics, being through multiple sites of cellular toxicity rather than through specific molecular action, hence the risk of developing resistance through their use is hypothetical. Judicious use of topical antiseptics reduces bio-burden in non-healing wounds and can prevent progression to systemic infection and the need for systemic antibiotics, with their added risk of antimicrobial resistance [11]; [15].

Silver has been used as an antimicrobial for centuries in many formulations [13]. Ionised silver (Ag+) has both anti-inflammatory and antimicrobial properties, with a broad spectrum of antimicrobial action with no clinical reports of inducing resistant organisms which are human pathogens [11]; [16]–[21]. Ionic silver appears to be incorporated into the bacterial cell wall and bacterial DNA, thereby blocking vital metabolic processes and cell proliferation [18]; [22]. Several studies have investigated the effect and safety of silver in the treatment of venous leg ulcers [23]–[29].

Two systematic reviews [14]; [30] have not been supportive of the use of silver-impregnated dressings to control bioburden and improve chronic leg ulcer healing rates, however more recent systematic reviews and meta-analyses have found that silver dressings significantly reduce odour, improve pain-related symptoms, decrease wound exudate, and have a prolonged dressing wear time compared with alternative wound treatments [31]; [32]. Furthermore, an improved quality of life with no associated severe adverse events with use of the dressings was found in the latter meta-analysis. Although one systematic review, using meta-analysis with the end point of complete wound healing, judged that the evidence was inconsistent regarding the effects of silver-based dressings and topical agents on leg ulcer healing [33] two other meta-analyses came to the opposite conclusion, asserting that silver dressings were effective in promoting the wound healing process, and strengthened the proposition that silver-impregnated dressings can improve the short term healing of wounds and ulcers [32]; [34]. The inconsistent conclusions may partly be due to different silver-releasing profiles of the investigated dressings; hence evaluating one specific silver-releasing dressing may reveal a more accurate result.

Biatain Ag is a soft absorbent polyurethane foam dressing which contains silver ions as an integral part of its matrix. In the presence of wound exudates ionic silver is released to the wound bed. The performance of Biatain Ag has been evaluated in several RCTs which measured the reduction of the area of hard-to-heal venous leg ulcers, as an end point, when compared with non silver-containing comparators [24]; [27]; [35]; [36]. The objective of this new meta-analysis of these RCTs was to examine the effect of Biatain Ag dressings in the management of hard-to-heal venous leg ulcers.

## Methods

### Data Sources

**Figure 1:**
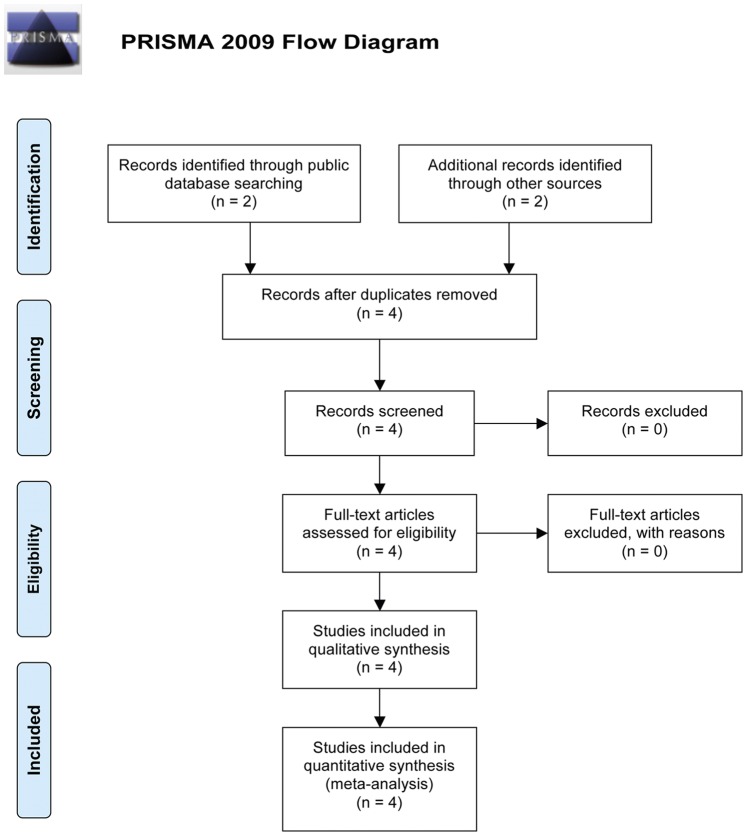
Flow chart of literature search.

Pubmed and the Cochrane Library were searched using the term ‘Biatain Ag’ or ‘Biatain Argent’ or ‘Biatain Plata’ or ‘Contreet’ without date restriction (Figure 1). An ‘in-house’ literature and knowledge’ database was also searched in using the same terms. For the present meta-analysis it was decided to include only data from RCTs. Of the studies found in which Biatain Ag dressings were used for the treatment of chronic leg ulcers; only four were RCTs [24]; [27]; [35]; [36]. In two of the RCTs [24]; [27] the dressing was Contreet® (a previous version of Biatain Ag with identical silver content and release), and in the two remaining studies the dressing was Biatain Ag, which is the currently used name for the product. None of the included data sets were overlapping.

The types of chronic leg ulcers, comparator dressings and methods used for ulcer area measurement are shown in Table 1. All subjects had leg ulcers that exhibited delayed healing (defined as clinical signs of infection (exudates, pain, discoloration, odor) and/or less than 20% ulcer size reduction over 4 weeks). However the study by Münter et al. [27] differed from the three other studies in a number of ways: the types of wound aetiologies included burns, post-operative wounds, leg ulcers, pressure ulcers, diabetic foot ulcers and other wound types; furthermore, the treatment chosen for the comparator group was ‘local practice’, and included other active dressings; and finally, the measurement of ulcer sizes was based on one axis measurement only. In the meta-analysis it was decided, for these reasons, to exclude patients from the comparator group who had been treated with active dressings or gauze, and include only venous or mixed-aetiology leg ulcers in the data set. The axis-based measurement of ulcer areas was assumed not to be critical for the meta-analysis as the primary outcome was based on relative reduction only. Compression was part of the treatment in both treatment groups in all 4 RCTs. An overview of the subjects included in the analysis and their baseline characteristics are shown in Table 2.

**Table 1:** Data sources considered for inclusion in the meta-analysis.

Studies^1^	Ulcer types^2^	Comparator	Ulcer area measurements
Jørgensen et al. (2005)	Venous/arterial ulcers	Foam dressing (Allevyn)	Planimetry only
Münter et al. (2006)	Venous, mixed, arterial, diabetic and pressure ulcers	Local best practice^3^	Axis based
Humbert et al. (2006)	Venous, mixed	Calcium alginate dressing (Algosteril)	Planimetry and axis based
Senet et al. (2013)	Venous	Foam dressing (Biatain)	Planimetry and axis based

1 All studies were multinational except Humbert et al. which was a French study.^ 2^Only subjects with venous or mixed ulcer aetiologies were selected for the meta-analysis.^3^Local Best Practice included foams/alginates (53%), hydrocolloids (12%), gauze (3%), silver dressings (17%), other microbial dressings (9%) and other active dressings (6%).

**Table 2:** Numbers and baseline characteristics of patients included in the meta-analysis.

Studies	Subjects in trial	Subjects included in analysis (%)	Reason for exclusion	Gender^1^, female (%)	Age^2^, mean (SD)	Baseline ulcer area^3^ (cm^2^), mean (SD)	Baseline ulcer age^4^ (years), mean (SD)
Jørgensen et al.(2005)	129	129 (100)	No exclusions	82 (63.6)	71.6 (12.4)	10.1 (9.8)	2.7 (4.2)
Münter et al. (2006)	619	315 (51)	Ulcer types other than venous or mixed. Active comparators or gauze	226 (71.7)	70.9 (12.5)	38.3 (69.6)	2.7 (5.0)
Humbert et al. (2006)	80	60 (75)	Ulcer type other thanvenous or mixed	41 (68.3)	74.4 (10.5)	13.2 (11.1)	1.9 (3.5)
Senet et al. (2013)	181	181 (100)	No exclusions	97 (53.6)	73.5 (12.2)	15.0 (13.7)	2.8 (4.7)
Total subjects	1009	685 (68)	–	446 (65.1)	–	–	

Baseline differences across the studies:^1^(p = 0.001), the fraction of females in the study by Senet et al. is substantially smaller. ^2^(p = 0.07) no significant difference of patient age, ^3^(p<0.0001), significant larger ulcers were included in the study by Münter et al, ^4^(p = 0.002), the study by Humbert et al. included somewhat younger ulcers.

The remaining products were considered sufficiently homogeneous to be used as a single class of comparators in the control arm Table 1.

### Meta-analysis (Statistical Pooling) and Statistical Methods

The baseline characteristics and measurement of ulcer area, at four weeks, were available in all included data sets and were selected as primary evaluation points in the meta-analysis. The meta-analysis was carried out on the 4 RCTs with the following outcomes:

- relative reduction of ulcer area over 4 weeks
- response rate; defined as the proportion of subjects with a relative ulcer area reduction of ≥40% at 4 weeks (indicative of a favorable healing prognosis [37])
- complete healing (healers), defined as the proportion of subjects with a healed ulcer at 4 weeks.

The patients’ demographics and baseline characteristics were summarized by treatment group using descriptive statistics. Baseline differences between studies were evaluated using Wilcoxon and Fishers exact test. The relative reduction of ulcer area over 4 weeks was analyzed by an ANCOVA model with baseline area and age as fixed effects, as these parameters were available for all studies. Responders and healers were analyzed by logistic regression. Treatment effects and the difference between these were estimated by the least-square means (LSMeans) extracted from the model, including confidence intervals and p-values for the treatment effect differences. The level for acceptance of statistical significance was set at 5%. Withdrawals were not excluded, provided that they had a statistically significant p-value (<0.05) of the ulcer area at baseline and at week 4. The use of last observation carried forward (LOCF; European Medicines Agency guideline, 2009), to impute missing values, was restricted to a few cases where an ulcer area at week 4 was missing while an area at week 3 was present. Analyses were performed using the SAS version 9.2 statistical package and the PRISMA guideline [38] was followed for the analyses.

### Sensitivity Analysis

A sensitivity analysis was conducted to evaluate the robustness of the results when the weight of the RCT data from Münter et al. [27] were downgraded by roughly 40, 60, 80, and 100%, corresponding to the sample sizes of the RCTs by Senet el al. [36] (N = 181), Jørgensen et al. [24] (N = 129), Humbert et al. [35] (N = 60) and finally no weight at all (N = 0).

### Ethic Statement

No ethic approval was obtained for the study as the analyses were conducted on already published data sets.

## Results

The total number of subjects in the combined four RCTs was 1055. However, 370 subjects were excluded from the analysis because they had ulcer types other than chronic venous/mixed-aetiology leg ulcers, or if they had been treated with an active comparator or gauze. The mean age within the four studies was 72.8 years and the average ulcer area in three of the four studies was in the range of 10–15 cm^2^, whereas it was 38 cm^2^ in the fourth study. The relative reduction of ulcer area at week 4 is shown study-wise and for the whole data set in Table 3.

**Table 3:** Percent relative reduction of ulcer area at week 4 for each study and the whole data set.

Studies	Comparator (SD)	Experimental (SD)	All (SD)
Jørgensen et al. (2005)	30.9 (41.5)	39.9 (37.7)	35.5 (39.7)
Münter et al. (2006)	26.6 (50.7)	49.8 (36.2)	41.3 (43.5)
Humbert et al. (2006)	12.9 (48.7)	29.8 (38.7)	21.1 (44.6)
Senet et al. (2013)	27.5 (37.0)	35.4 (35.2)	31.3 (36.3)
All	26.3 (45.0)	43.5 (37.0)	36.0 (41.6)

**Figure 2:**
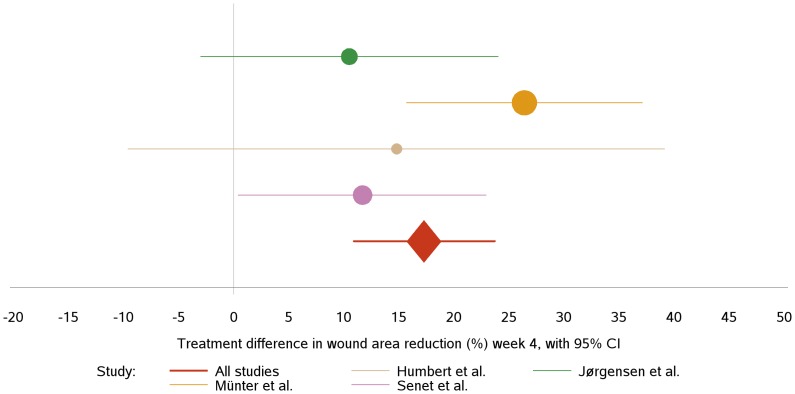
Forest plot showing the estimated treatment differences defined by percentage relative reduction. The solid vertical line represents a treatment difference of zero. The confidence intervals (95%) are illustrated by the length of the horizontal lines. The sizes of the filled circles are adjusted to the size of the corresponding study.

The treatment effects and the differences between these was estimated by least square means (LSMeans) extracted from the ANCOVA model, including confidence intervals and p-values for the treatment effect differences. The results are depicted in a Forest plot in Figure 2 and listed in.

Table 4. The relative reductions are markedly lower overall in the study published by Humbert [35] and neither this, nor the study by Jørgensen et al. [24], showed statistically significant treatment effects. The study by Senet et al. [36] showed a significant treatment difference in favour of Biatain Ag (p = 0.043). The combined results are clearly influenced by the Münter et al. study [27]. However, it should be noted that the estimated treatment differences point in the same direction and are within the range of 15–25%. This latter study [27] was downgraded to assess the sensitivity of the meta-analysis to the presence of this dominant study (Table 5). From 0 to 100% exclusion of the study the effect of Biatain Ag was lowered from a 17 percent point relative reduction in ulcer area at week 4 (p<0.0001) to an 11 percent point. Nevertheless, the relative reduction in ulcer area was still significantly higher (p = 0.01) in the Biatain Ag group after 100% exclusion of this study [27].

**Table 4:** Total and study wise treatment effects.

Studies	Control		Experimental		Treatment diff. CI	p-value
	n	LSMeans	n	LSMeans		
Jørgensen et al. (2005)	61	34.16	64	44.61	10.45 [−3.07; 23.96]	0.1285
Münter et al. (2006)	115	27.15	200	53.54	26.39 [15.70; 37.08]	<0.0001
Humbert et al. (2006)	30	11.24	28	26.03	14.79 [−9.55; 39.13]	0.2283
Senet et al. (2013)	84	29.27	77	40.92	11.65 [0.38; 22.92]	0.0428
All	290	25.46	369	42.78	17.31 [10.90; 23.73]	<0.0001

Treatment effects are estimated by least square means (extracted from the ANCOVA model) with confidence intervals and p-values for each study and the whole data set.

**Table 5:** The relative reduction in ulcer area when downgrading the weight of the study by Münter et al.

Reduced Münter et al study size,% size reduction (N)	Reduced meta-analysissize, N	Overall Biatain Ag effect in %-point			
		Estimate	Lower CL	Upper CL	P-value
100 (0)	370	−10.74	−18.89	−2.58	0.01001
81 (60)	430	−12.62	−18.72	−6.51	0.00006
59 (129)	499	−14.32	−20.54	−8.10	0.00001
43 (181)	551	−15.34	−21.63	−9.05	<0.00001
0 (315)	685	−17.31	−23.73	−10.90	<0.00001

The proportion of responders (patients with a relative reduction in ulcer area ≥40%) was 52% in the Biatain Ag group and 37% in the comparator group; with a significant treatment effect in favor of Biatain Ag (p<0.001). The fraction of ulcers healed was also significant higher for subjects treated with Biatain Ag (12%) compared with the comparator group (6%; p<0.002).

## Discussion

Antiseptics have been judged, mainly from experimental studies, as being toxic to healing tissues in chronic open wounds healing by secondary intention, unless used in dilute forms. However, there is little clinical evidence to support this. It is critical that use of these antiseptics, as topical antimicrobials, is not confused with the use of topical disinfectants which have been shown to be more cytotoxic in experimental studies, although some clinicians anecdotally argue that their use in undiluted forms allows efficient wound bed preparation of colonized wounds prior to skin grafting [7]–[12].

The rise of antibiotic resistant organisms, meticillin-resistant staphylococci in particular, together with the acute decline of new antibiotic research and introduction, is another major reason to revisit the use of topical antiseptics, which includes the use of silver dressings [39]. In addition to controlling the progression of colonisation, through reduction of bioburden, antiseptic dressings can reduce the risk of biofilm formation; aid in debridement; prepare the wound bed prior to healing; and act in infection prevention and control [40]. A further development, of increasing concern, is a theoretical concept that antiseptics may lead to the development of widespread antimicrobial resistance of human pathogenic organisms, not only to antiseptics but also to antibiotics. No such resistance in human pathogens has been seen in two millennia of silver use, and there is little evidence to support this hypothetical risk [16]; [17]; [19]; [20].

A further block to the use of antiseptic dressings has been their perceived cost, particularly those containing silver or presentations in complex dressings. Procurement managers are always ready to quote the findings of meta-analyses which are used to give “gold standard” of evidence based medicine. The Cochrane Collaboration has made several analyses of wound healing methodologies which have shown little or no evidence on level 1A for the use of different types of products and procedures [40]–[44]. This makes it problematic to organize a treatment plan and to produce guidelines for practitioners. The VULCAN study examined the role of several silver dressings in clean, healing venous ulcers; this was inappropriate and included no microbiology [40]; [41]; [45]. Antimicrobial silver dressings are an established, effective element of wound care which cannot be ignored.

Biatain Ag is a dressing which has the dual action of being a foam, which can handle exudate, and containing silver, as an antimicrobial. The efficacy and safety of Biatain Ag have been evaluated in four independent RCTs [24]; [27]; [35]; [36] of various size, each showing varying degree of efficacy. In the present study a meta-analysis was conducted on the combined data set from the four studies revealing a superior performance of Biatain Ag with a significant treatment effect (p<0.0001). A significantly better performance using Biatain Ag dressings was also seen in terms of the responder rate (p<0.001) and healing rate (p = 0.002).

Limitations of the study: Wound care is not standardized or consistent in clinical practice where regional and national differences obviously exist. The study design of the four included RCTs also differed as well as the study population. The analysis of baseline parameters showed that ulcer size and age varied between studies [27]; [35]. The outcome of the meta-analysis was limited to those parameters available for all four trials; e.g. the analysis was limited to 4 weeks follow-up period even though two of the RCTs included data for a 6-weeks follow-up period. The results of the meta-analysis are consistent although the relatively large number of patients included in the study by Münter et al. [27] influence the degree of significance which was corroborated by the sensitivity analysis.

In the RCTs considered in this meta-analysis of the Biatain Ag dressing the pooled estimate gives a significant statistical evidence to support its use as an antimicrobial dressing in the treatment of hard-to-heal venous leg ulcers.

## Supporting Information

Checklist S1 (DOCX)

## References

[1] Mekkes JR, Loots MA, van der Wal AC, Bos JD (2003) Causes, investigation and treatment of leg ulceration. Br J Dermatol 148: 388–401. 12653729 10.1046/j.1365-2133.2003.05222.x

[2] Baker SR, Stacey MC, Singh G, Hoskin SE, Thompson PJ (1992) Aetiology of chronic leg ulcers. Eur J Vasc Surg 6: 245–251. 1592127 10.1016/s0950-821x(05)80313-5

[3] Cornwall JV, Dore CJ, Lewis JD (1986) Leg ulcers: epidemiology and aetiology. Br J Surg 73: 693–696. 3756430 10.1002/bjs.1800730905

[4] Margolis DJ, Bilker W, Santanna J, Baumgarten M (2002) Venous leg ulcer: incidence and prevalence in the elderly. J Am Acad Dermatol 46: 381–386. 11862173 10.1067/mjd.2002.121739

[5] Ayello EA, Cuddigan JE (2004) Conquer chronic wounds with wound bed preparation. Nurse Pract 29: 8–25. 10.1097/00006205-200403000-00002 15021498

[6] Davies CE, Hill KE, Newcombe RG, Stephens P, Wilson MJ, et al (2007) A prospective study of the microbiology of chronic venous leg ulcers to reevaluate the clinical predictive value of tissue biopsies and swabs. Wound Repair Regen 15: 17–22. 17244315 10.1111/j.1524-475X.2006.00180.x

[7] Dissemond J, Assadian O, Gerber V, Kingsley A, Kramer A, et al (2011) Classification of wounds at risk and their antimicrobial treatment with polihexanide: a practice-oriented expert recommendation. Skin Pharmacol Physiol 24: 245–255. 21508658 10.1159/000327210

[8] Gray D, Barrett S, Battacharya M, Butcher M, Enoch S, et al (2010) PHMB and its potential contribution to wound management. Wounds 6: 40–46.

[9] Leaper D, Harding K (2010) Antimicrobials and Antiseptics. Journal of Wound Technology 7: 34–35.

[10] Leaper D (2011) Topical antiseptics in wound care: time for reflection. Int Wound J 8: 547–549. 22074499 10.1111/j.1742-481X.2011.00872.x PMC7950654

[11] Leaper DJ (2006) Silver dressings: their role in wound management. Int Wound J 3: 282–294. 17199764 10.1111/j.1742-481X.2006.00265.x PMC7951582

[12] Leaper DJ, Schultz G, Carville K, Fletcher J, Swanson T, et al (2012) Extending the TIME concept: what have we learned in the past 10 years. Int Wound J 9 Suppl 21–19. 10.1111/j.1742-481X.2012.01097.x PMC7950760 23145905

[13] International consensus. Appropriate use of silver dressings in wounds. An expert working group consensus. London: Wounds International. 2012. Available: http://www.woundsinternational.com/pdf/content_10381.pdf. Accessed 2013 May 23.

[14] Vermeulen H, van Hattem JM, Storm-Versloot MN, Ubbink DT (2007) Topical silver for treating infected wounds. Cochrane Database Syst Rev CD005486. 10.1002/14651858.CD005486.pub2 17253557

[15] White R, Cutting K (2006) Exploring the effects of silver in wound management - what is optimal? Wounds 18: 307–314.

[16] Cutting K, White R, Edmonds M (2007) The safety and efficacy of dressings with silver - addressing clinical concerns. Int Wound J 4: 177–184. 17651232 10.1111/j.1742-481X.2007.00338.x PMC7951405

[17] Ip M, Lui SL, Chau SS, Lung I, Burd A (2006) The prevalence of resistance to silver in a Burns unit. J Hosp Infect 63: 342–344. 16650502 10.1016/j.jhin.2006.02.005

[18] Lansdown AB (2002) Silver. I: Its antibacterial properties and mechanism of action. J Wound Care 11: 125–130. 11998592 10.12968/jowc.2002.11.4.26389

[19] Lansdown AB, Williams A (2007) Bacterial resistance to silver in wound care and medical devices. J Wound Care 16: 15–19. 17334141 10.12968/jowc.2007.16.1.26983

[20] Percival SL, Bowler P, Woods EJ (2008) Assessing the effect of an antimicrobial wound dressing on biofilms. Wound Repair Regen 16: 52–57. 18211579 10.1111/j.1524-475X.2007.00350.x

[21] Robson MC (1997) Wound infection. A failure of wound healing caused by an imbalance of bacteria. Surg Clin North Am 77: 637–650. 9194884 10.1016/s0039-6109(05)70572-7

[22] Hermans MH (2007) Silver-containing dressings and the need for evidence. Adv Skin Wound Care 20: 166–173. 17473723 10.1097/01.ASW.0000262712.05035.61

[23] Harding K, Gottrup F, Jawien A, Mikosinski J, Twardowska-Saucha K, et al (2011) A prospective, multi-centre, randomised, open label, parallel, comparative study to evaluate effects of AQUACEL((R)) Ag and Urgotul((R)) Silver dressing on healing of chronic venous leg ulcers. Int Wound J 9: 285–294. 22066961 10.1111/j.1742-481X.2011.00881.x PMC7950382

[24] Jorgensen B, Price P, Andersen KE, Gottrup F, Bech-Thomsen N, et al (2005) The silver-releasing foam dressing, Contreet Foam, promotes faster healing of critically colonised venous leg ulcers: a randomised, controlled trial. Int Wound J 2: 64–73. 16722854 10.1111/j.1742-4801.2005.00084.x PMC7951198

[25] Karlsmark T, Agerslev RH, Bendz SH, Larsen JR, Roed-Petersen J, et al (2003) Clinical performance of a new silver dressing, Contreet Foam, for chronic exuding venous leg ulcers. J Wound Care 12: 351–354. 14601228 10.12968/jowc.2003.12.9.26534

[26] Lazareth I, Ourabah Z, Senet P, Cartier H, Sauvadet A, et al (2007) Evaluation of a new silver foam dressing in patients with critically colonised venous leg ulcers. J Wound Care 16: 129–132. 17385590 10.12968/jowc.2007.16.3.27015

[27] Munter KC, Beele H, Russell L, Crespi A, Grochenig E, et al (2006) Effect of a sustained silver-releasing dressing on ulcers with delayed healing: the CONTOP study. J Wound Care 15: 199–206. 16711173 10.12968/jowc.2006.15.5.26909

[28] Vanscheidt W, Lazareth I, Routkovsky-Norval C (2003) Safety evaluation of a new ionic silver dressing in the management of chronic ulcers. Wounds 15: 371–378.

[29] Wunderlich U, Orfanos CE (1991) Treatment of venous ulcera cruris with dry wound dressings. Phase overlapping use of silver impregnated activated charcoal xerodressing. Hautartz 42: 446–450. 1938398

[30] Storm-Versloot MN, Vos CG, Ubbink DT, Vermeulen H (2010) Topical silver for preventing wound infection. Cochrane Database Syst Rev CD006478. 10.1002/14651858.CD006478.pub2 20238345

[31] Lo SF, Hayter M, Chang CJ, Hu WY, Lee LL (2008) A systematic review of silver-releasing dressings in the management of infected chronic wounds. J Clin Nurs 17: 1973–1985. 18705778 10.1111/j.1365-2702.2007.02264.x

[32] Lo SF, Chang CJ, Hu WY, Hayter M, Chang YT (2009) The effectiveness of silver-releasing dressings in the management of non-healing chronic wounds: a meta-analysis. J Clin Nurs 18: 716–728. 19239539 10.1111/j.1365-2702.2008.02534.x

[33] Chambers H, Dumville JC, Cullum N (2007) Silver treatments for leg ulcers: a systematic review. Wound Repair Regen 15: 165–173. 17352747 10.1111/j.1524-475X.2007.00201.x

[34] Carter MJ, Tingley-Kelley K, Warriner RA III (2010) Silver treatments and silver-impregnated dressings for the healing of leg wounds and ulcers: a systematic review and meta-analysis. J Am Acad Dermatol 63: 668–679. 20471135 10.1016/j.jaad.2009.09.007

[35] Humbert P, Zuccarelli F, Debure C, Vendeaud Busquet F, Bressieux JM, et al (2006) Ulcères de jambe présentant des signes locaux d'infection: intérêt du pansement Biatain Argent [Leg ulcers showing local signs of infection: benefits of the Biatain Argent dressing]. Journal des plaies et cicatrisations 52: 41–47.

[36] Senet P, Bause R, Jorgensen B, Fogh K (2013) Clinical efficacy of a silver-releasing foam dressing in venous leg ulcer healing - a randomized controlled trial. Int Wound J Epub ahead of print. 10.1111/iwj.12022 PMC7950477 23374589

[37] Flanagan M (2003) Wound measurement: can it help us to monitor progression to healing? J Wound Care 12: 189–194. 12784601 10.12968/jowc.2003.12.5.26493

[38] Moher D, Altman DG, Liberati A, Tetzlaff J (2011) PRISMA statement. Epidemiology 22: 128. 21150360 10.1097/EDE.0b013e3181fe7825

[39] Tackling antibacterial resistance in Europe. European Academies Science Advisory Council. Royal Society. London. 2007. Available: http://www.easac.eu/fileadmin/PDF_s/reports_statements/Tackling.pdf. Accessed 2013 May 23.

[40] Leaper D, Drake R (2011) Should one size fit all? An overview and critique of the VULCAN study on silver dressings. Int Wound J 8: 1–4. 21251222 10.1111/j.1742-481X.2010.00766.x PMC7950782

[41] Gottrup F, Apelqvist J (2010) The challenge of using randomized trials in wound healing. Br J Surg 97: 303–304. 20140951 10.1002/bjs.7030

[42] Leaper D (2009) Evidence-based wound care in the UK. Int Wound J 6: 89–91. 19432658 10.1111/j.1742-481X.2009.00581.x PMC7951697

[43] Leaper D (2009) Cochrane: hands off wound care. Int Wound J 6: 309–310.

[44] Gottrup F, Apelqvist J (2012) Present and new techniques and devices in the treatment of DFU: a critical review of evidence. Diabetes Metab Res Rev 28 Suppl 164–71. 22271726 10.1002/dmrr.2242

[45] Michaels JA, Campbell WB, King BM, Macintyre J, Palfreyman SJ, et al.. (2009) A prospective randomised controlled trial and economic modelling of antimicrobial silver dressings versus non-adherent control dressings for venous leg ulcers: the VULCAN trial. Health Technol Assess 13: 1–114, iii. 10.3310/hta13560 19939335

